# Inhibition of Nox2 Oxidase Activity Ameliorates Influenza A Virus-Induced Lung Inflammation

**DOI:** 10.1371/journal.ppat.1001271

**Published:** 2011-02-03

**Authors:** Ross Vlahos, John Stambas, Steven Bozinovski, Brad R. S. Broughton, Grant R. Drummond, Stavros Selemidis

**Affiliations:** 1 Department of Pharmacology, The University of Melbourne, Melbourne, Victoria, Australia; 2 School of Medicine, Deakin University, Geelong, Victoria, Australia; 3 Department of Pharmacology, Monash University, Melbourne, Victoria, Australia; St Jude Children's Research Hospital, United States of America

## Abstract

Influenza A virus pandemics and emerging anti-viral resistance highlight the urgent need for novel generic pharmacological strategies that reduce both viral replication and lung inflammation. We investigated whether the primary enzymatic source of inflammatory cell ROS (reactive oxygen species), Nox2-containing NADPH oxidase, is a novel pharmacological target against the lung inflammation caused by influenza A viruses. Male WT (C57BL/6) and Nox2^−/y^ mice were infected intranasally with low pathogenicity (X-31, H3N2) or higher pathogenicity (PR8, H1N1) influenza A virus. Viral titer, airways inflammation, superoxide and peroxynitrite production, lung histopathology, pro-inflammatory (MCP-1) and antiviral (IL-1β) cytokines/chemokines, CD8^+^ T cell effector function and alveolar epithelial cell apoptosis were assessed. Infection of Nox2^−/y^ mice with X-31 virus resulted in a significant reduction in viral titers, BALF macrophages, peri-bronchial inflammation, BALF inflammatory cell superoxide and lung tissue peroxynitrite production, MCP-1 levels and alveolar epithelial cell apoptosis when compared to WT control mice. Lung levels of IL-1β were ∼3-fold higher in Nox2^−/y^ mice. The numbers of influenza-specific CD8+D^b^NP_366_+ and D^b^PA_224_+ T cells in the BALF and spleen were comparable in WT and Nox2^−/y^ mice. *In vivo* administration of the Nox2 inhibitor apocynin significantly suppressed viral titer, airways inflammation and inflammatory cell superoxide production following infection with X-31 or PR8. In conclusion, these findings indicate that Nox2 inhibitors have therapeutic potential for control of lung inflammation and damage in an influenza strain-independent manner.

## Author Summary

Influenza A virus pandemics are imminent and with emerging anti-viral resistance highlight an ongoing, urgent need for novel generic pharmacological strategies. Ideally these strategies should reduce both viral replication and lung inflammation, *irrespective of the infecting strain* by modulating the host immune response. An important paradigm strongly suggests that the lung damage arising from not only influenza A viruses but other pathogens including, but not restricted to, SARS, parainfluenza viruses, *human respiratory syncytial virus and Streptococcus pneumoniae* consists of an excessive host response characterised by a rapid, influx of inflammatory cells into the lungs leading to excessive reactive oxygen species (ROS) production. Our study demonstrates that the primary enzymatic source of inflammatory cell ROS, Nox2-containing NADPH oxidase, promotes airways inflammation to low and high pathogenicity influenza A virus infection and impedes with the host's ability to clear the virus. Thus, Nox2 inhibitors could be considered individually or in combination with current antiviral strategies for control of future influenza A virus pandemics.

## Introduction

The early host innate immune response directed against influenza A virus infection in the absence of pre-existing immunity is typically characterised by activation of airway epithelium and resident alveolar macrophages, and release of inflammatory mediators resulting in the trafficking of additional macrophages, neutrophils and T lymphocytes into the lung [1]. The recruitment of macrophages and neutrophils into the lung controls seasonal influenza virus and results in mild clinical symptoms. However, some pandemic influenza A viruses initiate an aggressive persistent trafficking of large numbers of inflammatory cells, which is now considered to be associated with lethal disease, culminating in severe lung injury as seen for H5N1 and 1918 pandemic influenza virus infection [2].

Recent evidence suggests that much of the acute lung injury caused by H5N1 can be attributed to excessive ROS production (i.e. oxidative stress) initiated by an overactive innate immune response [3], [4]. ROS including superoxide anion and its derivatives peroxynitrite (OONO^−^), hydrogen peroxide (H_2_O_2_) and hydroxyl radical (OH^.^) are indiscriminately toxic to cells when produced in excess and capable of regulating pro-inflammatory cytokine production. The cellular source of ROS is most likely to be infiltrating inflammatory cells, which on a cell-to-cell basis generate more ROS than any other cell type [5], [6]. Identification of the enzymatic sources of ROS may pave the way for therapies that combat the oxidative stress-dependent lung injury caused by influenza A virus infection.

A number of enzyme systems expressed in mammalian cells are capable of generating superoxide (for reviews see [5], [6]). However, NADPH oxidase is the primary source of superoxide production by inflammatory cells [5], [6]. The inflammatory cell NADPH oxidase enzyme consists of a number of protein subunits including the catalytic subunit Nox2, the smaller α-subunit, p22^phox^, as well as multiple regulatory subunits, including the organizer protein p47^phox^, the activator protein p67^phox^, p40^phox^ and the small G protein Rac1. Nox2 was recently shown to play a role in the clearance of influenza infection and in lung dysfunction [7]. However, it remains to be determined if Nox2 influences: (i) low and high pathogenicity influenza A virus infection, (ii) the infiltration of sub-populations of inflammatory cells into the airways, (iii) superoxide and peroxynitrite production by key inflammatory cells in the airways, (iv) alveolar epithelial cell apoptosis, (v) the levels of potential antiviral nitric oxide (NO) generated and (vi) crucial adaptive immune responses that clear influenza virus. Moreover, it is unknown if pharmacological inhibitors of Nox2 protect against influenza virus-induced airways inflammation and damage. The present study clearly shows that Nox2 is critical for the control of influenza infection and that inhibition of Nox2 may be a way of controlling future pandemics by modulating the host *instead* of the virus.

## Results

### Nox2^−/y^ mice show reduced BALF inflammation in response to X-31 virus

**Figure 1:**
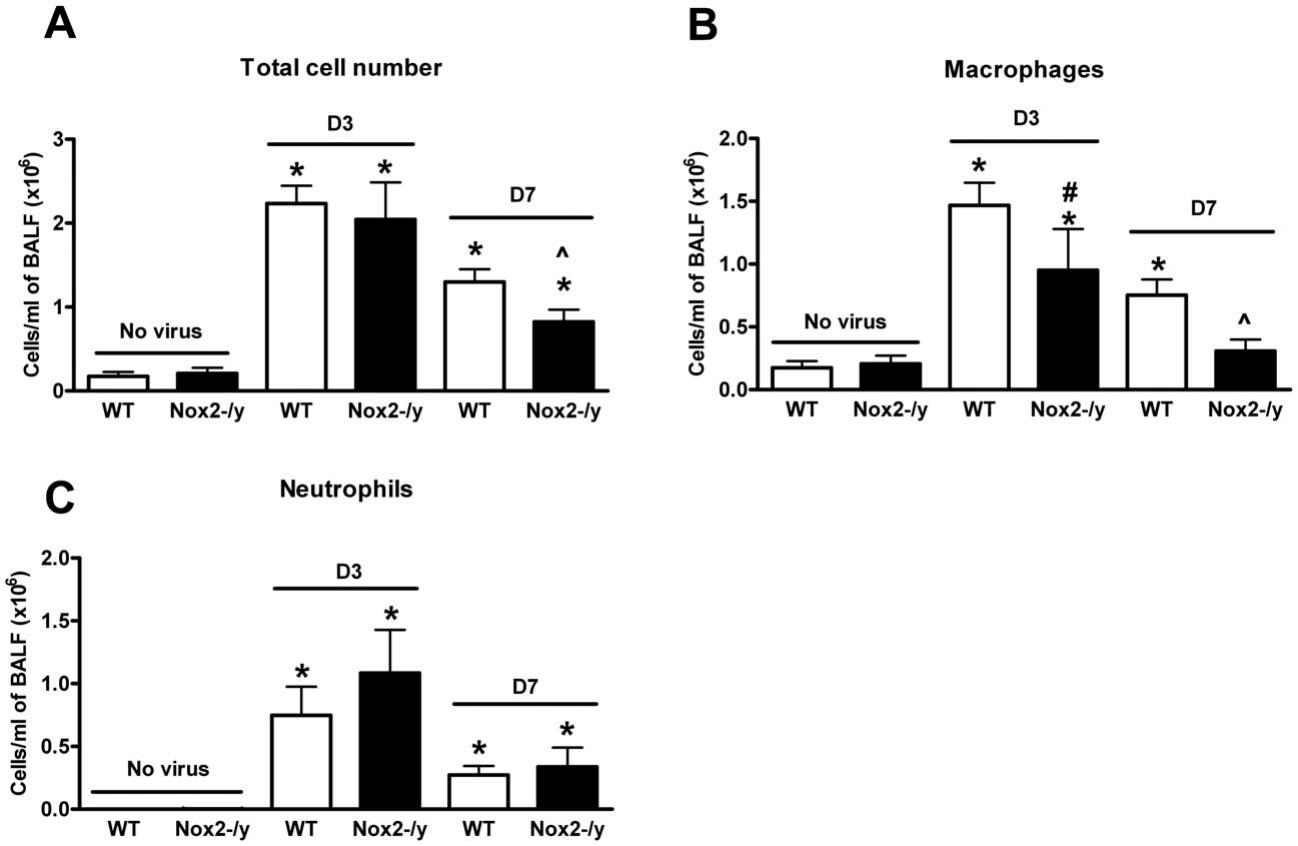
Effect of H3N2 (X-31) influenza A virus infection on BALF cellularity in wild type (WT) and Nox2^−/y^ mice. Mice were treated with 1×10^4^ PFU of the low virulence H3N2 strain of influenza A virus and the number of (A) total cells, (B) macrophages and (C) neutrophils counted in the BALF 3 and 7 days post infection. Data are shown as mean ± SD for 6–8 mice per group. *P<0.05 vs respective no virus group, #*P*<0.05 vs D3 X-31-treated WT mice mice, ∧*P*<0.05 vs D7 X-31-treated WT mice (ANOVA and Dunnett's *post hoc* test).

Naïve (i.e. no virus) Nox2^−/y^ mice had similar total cell numbers in the BALF to naïve WT mice (Figure 1A). We then analysed the extent of cellular infiltration in the BALF of WT and Nox2^−/y^ mice infected with X-31 virus 3 and 7 days post infection. Three days following infection, the cellular infiltrate in WT mice increased by ∼12.5 fold from basal levels (Figure 1A), which was similar in Nox2^−/y^ mice (Figure 1A). However, by Day 7 post infection, the total number of cells in Nox2^−/y^ mice was significantly lower (∼40%, *P*<0.05) when compared to WT controls (Figure 1A).

Close analyses of the infiltrating and resident populations by differential cell counting revealed that both 3 and 7 days post X-31 infection, BALF from Nox2^−/y^ mice contained significantly less (*P*<0.05) macrophages than WT mice (Figure 1B). There was a trend for neutrophil numbers to be higher in Nox2^−/y^ mice compared to WT mice infected with X-31 although this was not statistically significant (Figure 1C).

### Histological investigation of lung inflammation in X-31 infected Nox2^−/y^ mice

**Figure 2:**
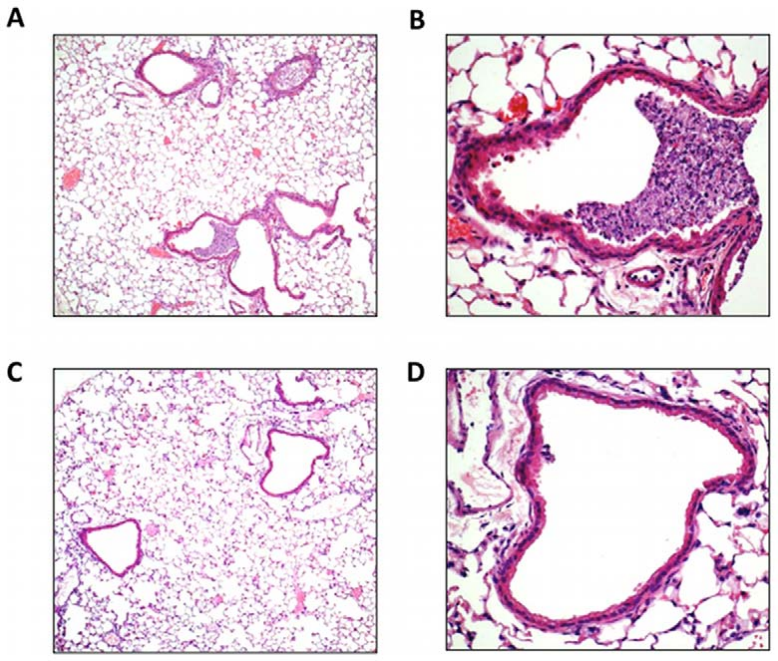
Lung histology in response to H3N2 (X-31) influenza A virus infection. (A–D) Hematoxylin and eosin stained paraffin sections of lungs from WT (A and B) and Nox2^−/y^ (C and D) mice obtained at Day 3. WT mice lungs had obvious peri-bronchial inflammation and prominent bronchial infiltrates consisting of mononuclear cells and neutrophils. In contrast, Nox2^−/y^ mice displayed considerably less bronchial cell infiltrates and peri-bronchial inflammation. Note, magnification of (A) and (C) is ×50 and (B) and (D) is ×200.

We also examined the degree of airway inflammation caused by X-31 in H&E stained lung sections from WT and Nox2^−/y^ mice killed at Day 3 post infection. We chose this time point to examine the lung inflammation as we have previously shown this to represent the peak of airways inflammation and viral load following X-31 virus infection [8]. Lungs from WT mice had obvious peri-bronchial inflammation and prominent bronchial infiltrates consisting of mononuclear cells and neutrophils (Figure 2A, B). In contrast, histological examination of Nox2^−/y^ mice showed considerably less bronchial cell infiltrate and peri-bronchial inflammation (Figure 2C, D).

### Nox2 is the primary source of superoxide and peroxynitrite production

**Figure 3:**
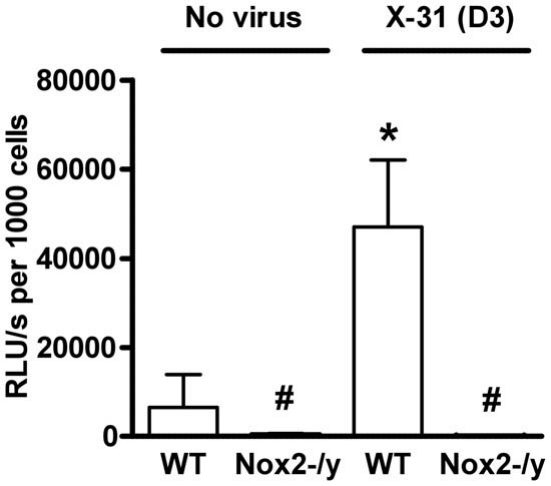
Superoxide production from BALF cells using L-O12-enhanced chemiluminescence. BALF cells obtained from H3N2 (X-31) influenza A virus-infected WT and Nox2^−/y^ mice 3 days post infection. Data are shown as mean ± SD relative light units per second (RLU/s) for 6–8 mice per treatment group. *P<0.05 vs WT no virus mice, #*P*<0.05 vs respective WT mice (ANOVA and Dunnett's *post hoc* test).

We examined basal and phorbol dibutyrate (PDB)-stimulated superoxide production by inflammatory cells in BALF of naïve and X-31-infected WT and Nox2^−/y^ mice. BALF cells isolated from naïve WT mice produced superoxide under basal conditions (data not shown), which was increased in the presence of PDB stimulation (Figure 3). In comparison, BALF cells isolated from naïve Nox2^−/y^ mice produced very little superoxide under both basal conditions (not shown) and following PDB stimulation (Figure 3). *Ex vivo* treatment of BALF cells with SOD (300U/ml) significantly reduced the chemiluminescence signal at 3 days following infection confirming that chemiluminescence resulted from superoxide detection (data not shown). BALF inflammatory cells from WT mice infected with X-31 produced significantly higher amounts (∼7-fold) of basal (not shown) and PDB-stimulated superoxide than BALF cells obtained from naïve mice (Figure 3). Again, superoxide production was virtually abolished in BALF cells isolated from lungs of Nox2^−/y^ mice (Figure 3).

**Figure 4:**
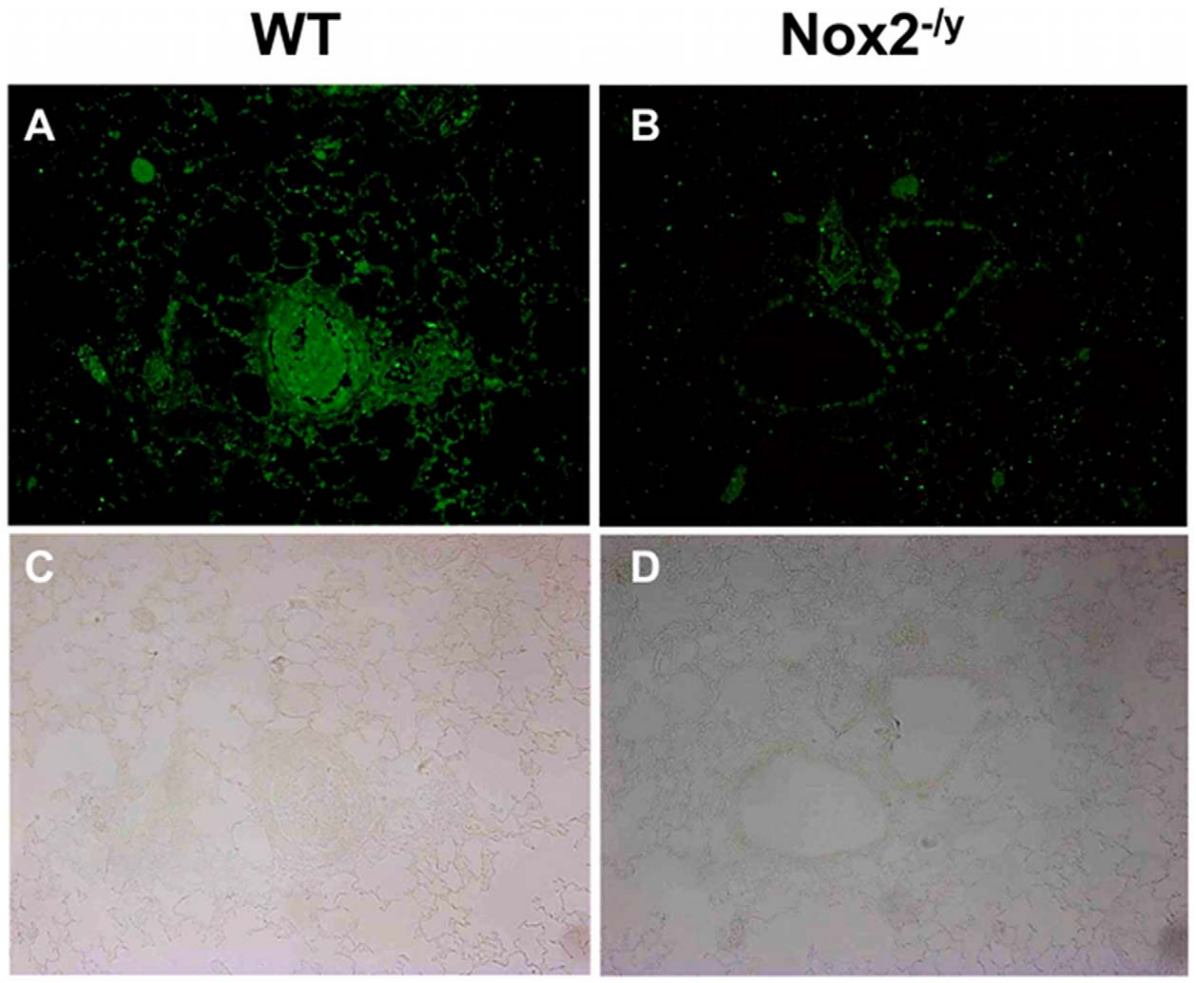
Peroxynitrite production in mouse lung infected with H3N2 (X-31) influenza A virus using 3-nitrotyrosine (3-NT) immunofluorescence. Representative sections of lung tissue obtained from WT (A) and Nox2^−/y^ mice (B) infected with X-31 were incubated with mouse monoclonal anti-3-nitrotyrosine antibody (1∶50) followed by biotinylated anti-mouse IgG reagent. (C and D) The same sections showing corresponding light microscope images. WT mice lung sections displayed strong immunofluorescence for 3-NT in the inflammatory cells that infiltrated the airways and in the alveolar tissue. In contrast, Nox2^−/y^ mice displayed markedly less immunofluorescence for 3-NT.

Superoxide gives rise to toxic reactive nitrogen species such as peroxynitrite when it reacts rapidly with NO. Thus, we examined 3-nitrotyrosine immunofluorescence as a measure of peroxynitrite production. Lung sections from WT mice infected with X-31 displayed strong immunofluorescence for 3-nitrotyrosine in the inflammatory cells that infiltrated the airways, as well as in the alveolar tissue (Figures 4A and C). By contrast, little immunofluorescence was observed in similar lung sections obtained from Nox2^−/y^ mice infected with X-31 (Figures 4B and D).

### Effect of Nox2 deletion on viral titers and body weight

**Figure 5:**
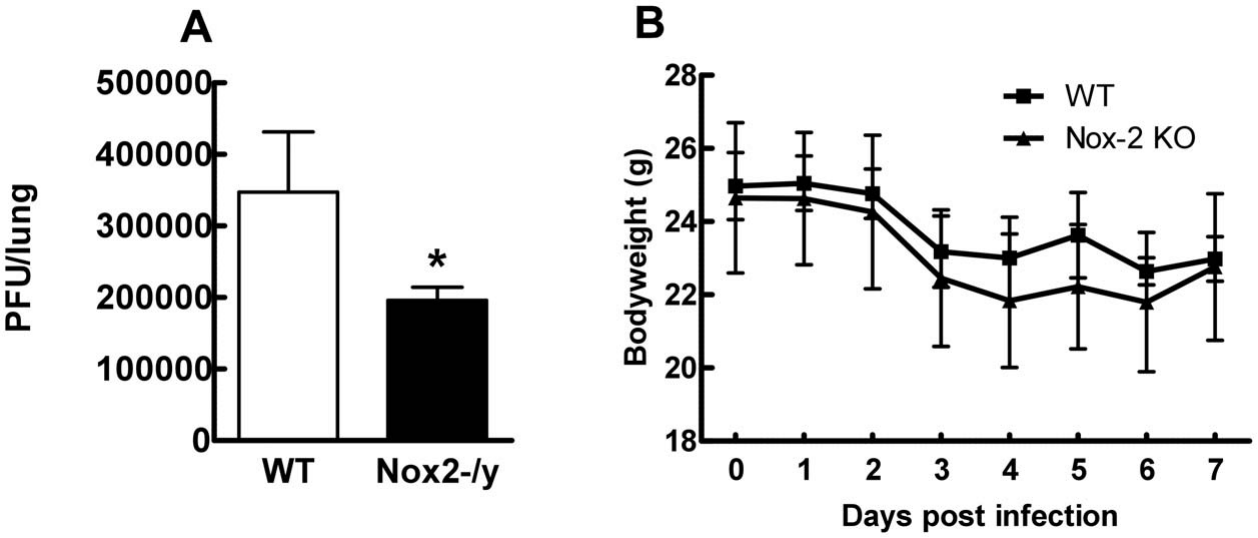
Effect of H3N2 (X-31) influenza A virus infection on viral titer and body weight in wild type (WT) and Nox2^−/y^ mice. Mice were treated with 1×10^4^ PFU of the low virulence H3N2 strain of influenza A virus and (A) viral titer determined 3 days post infection and (B) body weight recorded for up to 7 days post infection. Data are shown as mean ± SD for 6–8 mice per group. *P<0.05 vs WT mice (Students' unpaired *t* test).

To determine if reduced inflammation of Nox2^−/y^ mice correlated with lower levels of virus, we measured lung virus titers using the plaque assay. Lung virus titers were significantly lower (∼45%, *P*<0.05) in Nox2^−/y^ mice than WT mice at the day 3 time-point (Figure 5A). We explored whether Nox2 deletion had an impact on X-31-induced body weight loss. WT and Nox2^−/y^ mice lost similar amounts of weight following X-31 infection (Figure 5B).

### Determination of NO levels in BALF

We hypothesized that the enhanced clearance of virus from the lungs of Nox2^−/y^ mice could be attributed to an enhanced level of NO in the absence of superoxide. To address this we measured nitrite levels in BALF using the Griess reaction 3 days after X-31 infection. BALF from Nox2^−/y^ mice had similar levels of nitrite (26.42±4.37 µM), and thus NO, to WT mice (22.05±4.48 µM) (*P* = 0.08; n = 9).

### Nox2 and apoptosis

**Figure 6:**
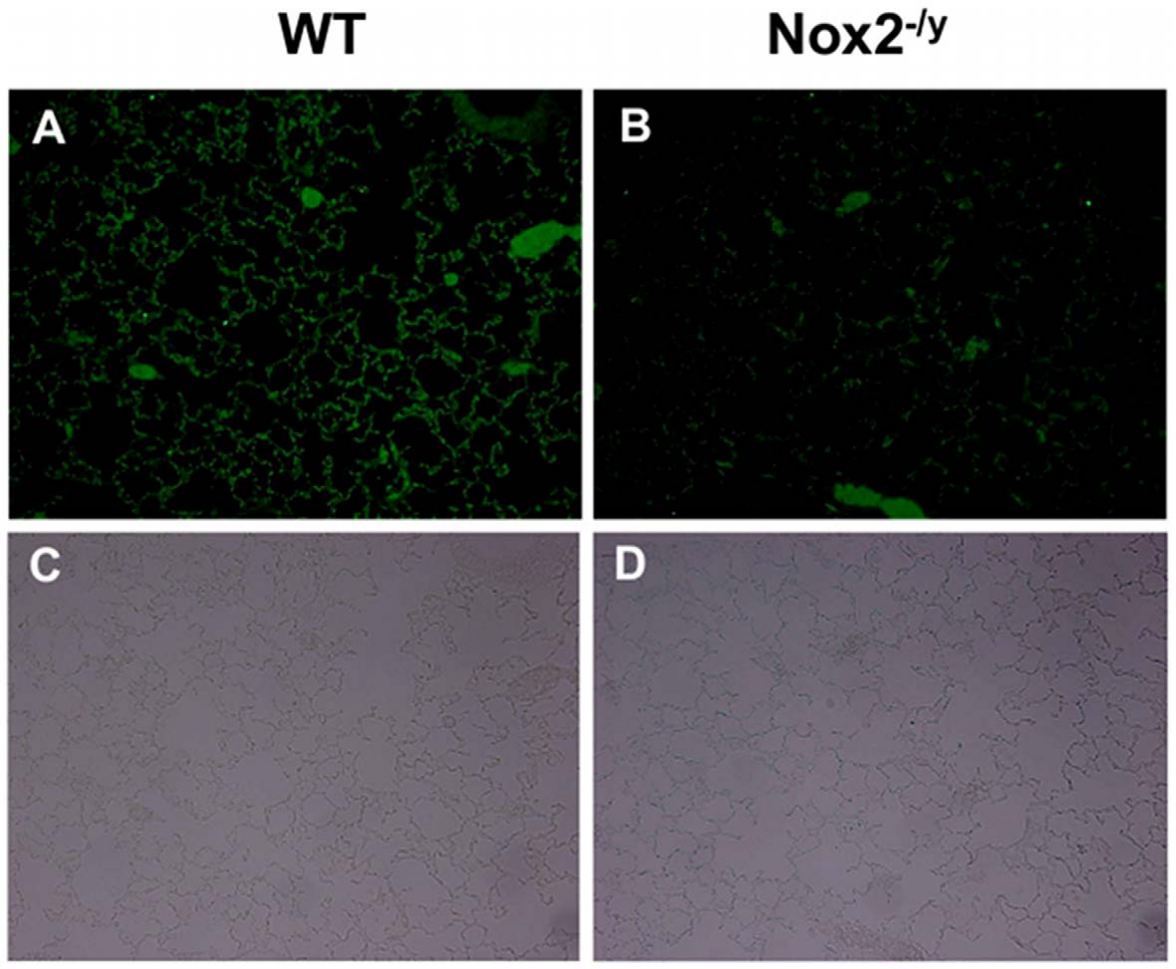
Cleaved caspase 3 immunofluorescence to assess lung alveolar epithelial apoptosis. Representative sections of lung tissue obtained from WT (A) and Nox2^−/y^ mice (B) infected with influenza A virus (H3N2; X-31) were incubated with rabbit polyclonal anti-cleaved caspase 3 antibody (1∶250) followed by goat anti-rabbit Alexa fluor 488 (Invitrogen; 1∶500) secondary antibody. (C and D) The same sections showing corresponding light microscope images. WT mice lung sections displayed strong immunofluorescence for cleaved caspase 3 in the alveolar tissue. In contrast, Nox2^−/y^ mice displayed markedly less immunofluorescence for cleaved caspase 3.

Accumulating evidence suggests that apoptosis of lung alveolar epithelial cells underlies alveolar injury and lung leakage, which are hallmarks of the acute lung injury/primary pneumonia following influenza A virus infection. Thus, we examined alveolar epithelial apoptosis using cleaved caspase 3 immunohistochemistry. Lung sections from WT mice infected with X-31 displayed strong immunofluorescence for cleaved caspase 3 in the alveolar tissue (Figures 6A and C). By contrast, there was strikingly very little immunofluorescence observed in similar lung sections obtained from Nox2^−/y^ mice infected with X-31 (Figure 6B and D).

### Examination of cytokine and chemokine expression in lungs

**Figure 7:**
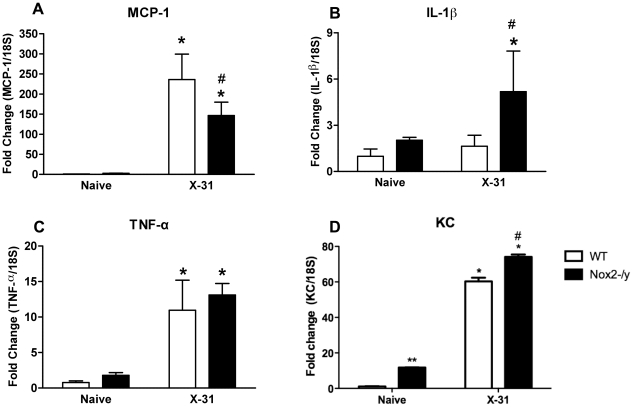
Assessment of lung cytokines levels. Effect of H3N2 (X-31) influenza A virus infection on (A) MCP-1, (B) IL-1β, (C) TNF-α and (D) KC mRNA expression in whole lung obtained from WT and Nox2^−/y^ mice 3 days post infection. Responses are shown as fold change relative to 18S. Data are shown as mean ± SD of 4 individual mice. WT and Nox2^−/y^ mice are shown as open and filled histograms, respectively. WT mice that did not receive H3N2 (i.e. naïve mice) were used as a control to demonstrate that virus infection caused an increase in MCP-1, IL-1β, TNF-α and KC. *P<0.05 vs respective naïve mice (ANOVA and Dunnett's *post hoc* test), #*P*<0.05 vs WT X-31-treated mice (ANOVA and Dunnett's *post hoc* test). ** P<0.05 vs WT naïve mice.

We hypothesised that the reduced macrophage infiltrate observed in Nox2^−/y^ mice following infection might be due to a decrease in chemokines responsible for macrophage recruitment. MCP-1 levels in WT lungs were significantly (*P*<0.05) increased 3 days post X-31 infection compared to naïve controls (Figure 7A). However, Nox2^−/y^ mice infected with X-31 virus had significantly ∼40% lower levels of MCP-1 compared to X-31-infected WT mice (Figure 7A). We also examined the expression of the cytokine IL-1β, which has been shown to enhance influenza virus clearance, protect from virus-induced mortality and stimulate neutrophil recruitment [9], [10]. Following infection with X-31 virus, the levels of IL-1β mRNA expression in Nox2^−/y^ mouse lungs were ∼3-fold higher than that in WT lungs (Figure 7B). The TNF-α expression level was also assessed given that it has been previously implicated in influenza immunopathology. There was no difference in TNF-α expression between Nox2^−/y^ mice and WT mice (Figure 7C). There were no significant differences in levels of MCP-1, IL-1β and TNFα between strains in naïve mice (Figure 7). We also explored whether there were changes in the neutrophil chemoattractant KC. QPCR revealed a small and significant increase in KC expression in both naïve and X-31 infected Nox2^−/y^ mice lungs compared to WT lungs, which supports the small, albeit insignificant increase in neutrophilia in the Nox2^−/y^ lungs (Figure 7D).

### Effect of Nox2 deletion on the magnitude and function of influenza-specific CD8+ T cell response

**Figure 8:**
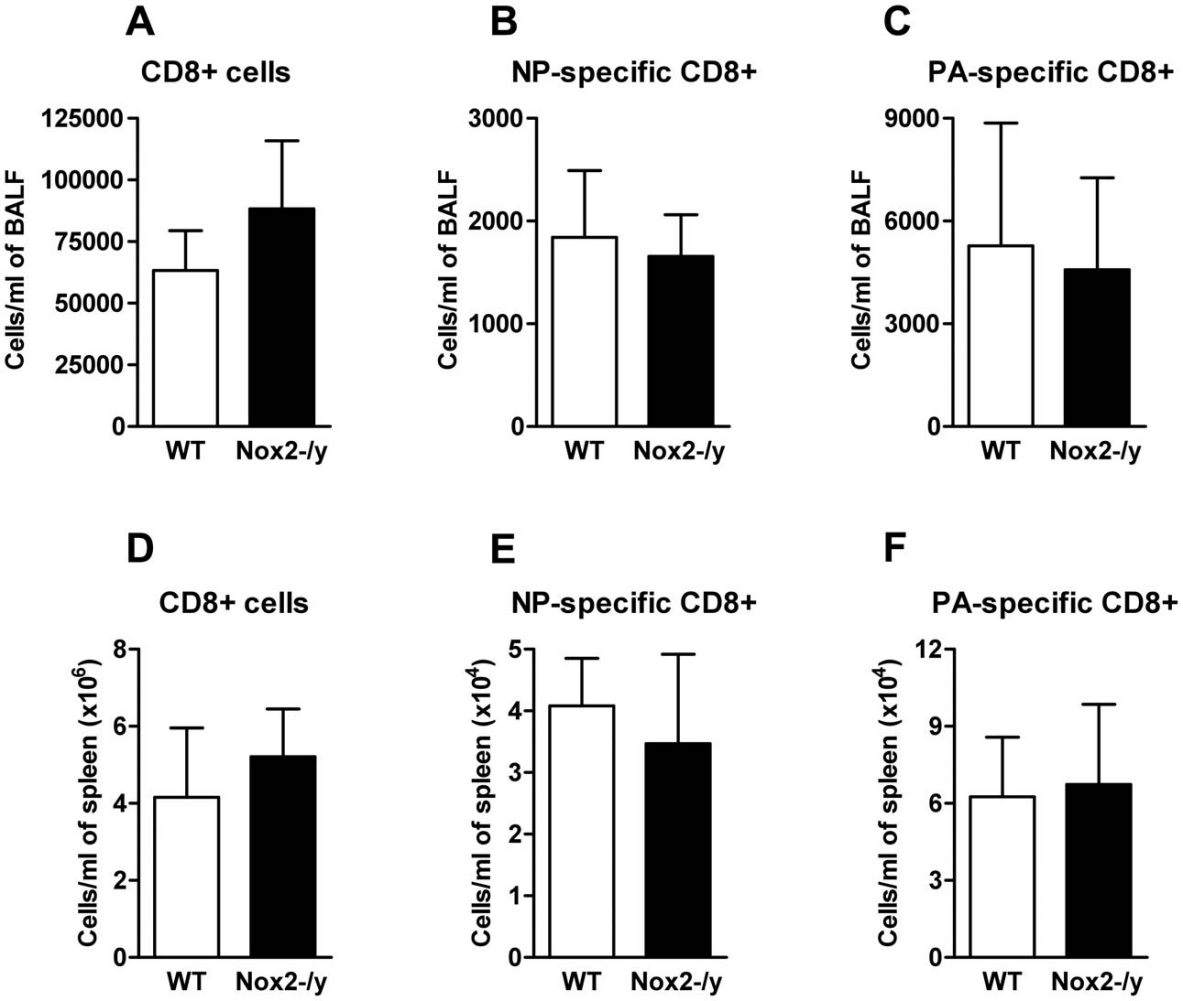
Number of the CD8+ T cell response. Enriched CD8+ T cells from BALF (A–C) or spleen (D–F) were obtained from influenza A virus (X-31)-infected WT and Nox2^−/y^ mice 7 days post infection. These were then stained with fluorescently labelled tetramer complexes specific for the two immunodominant CD8+ T cell epitopes (D^b^NP_366–372_ and D^b^PA_224–232_) and data analysed by flow cytometry. Data are shown as mean ± SD of 4 individual mice.

NADPH oxidase-deficient T cells have been previously shown to produce a Th1 skewed cytokine profile following TCR stimulation with cross-linking antibody [11]. Therefore we investigated the effect of Nox2^−/y^ deficiency on the magnitude and polyfunctionality of two immunodominant CD8+ T cell specificities. Tetramer staining of the BALF (Figure 8A–C) and spleen (Figure 8D–F) showed no significant differences in CD8+ T cell numbers between WT and Nox2^−/y^ animals. Furthermore influenza-specific CD8+D^b^NP_366_+ and D^b^PA_224_+CD8+ T cells were stimulated *in vitro* with relevant peptide and cytokine expression (IFN-γ, TNF-α and IL-2) detected using intracellular cytokine staining. There were no differences in cytokine expression observed between Nox2^−/y^ and WT animals (data not shown). This suggests that absence of Nox2 does not affect CD8+ T cell effector function.

### Pharmacological inhibition of Nox2 in vivo protects against X-31 and PR8 influenza virus-induced lung inflammation and damage

**Figure 9:**
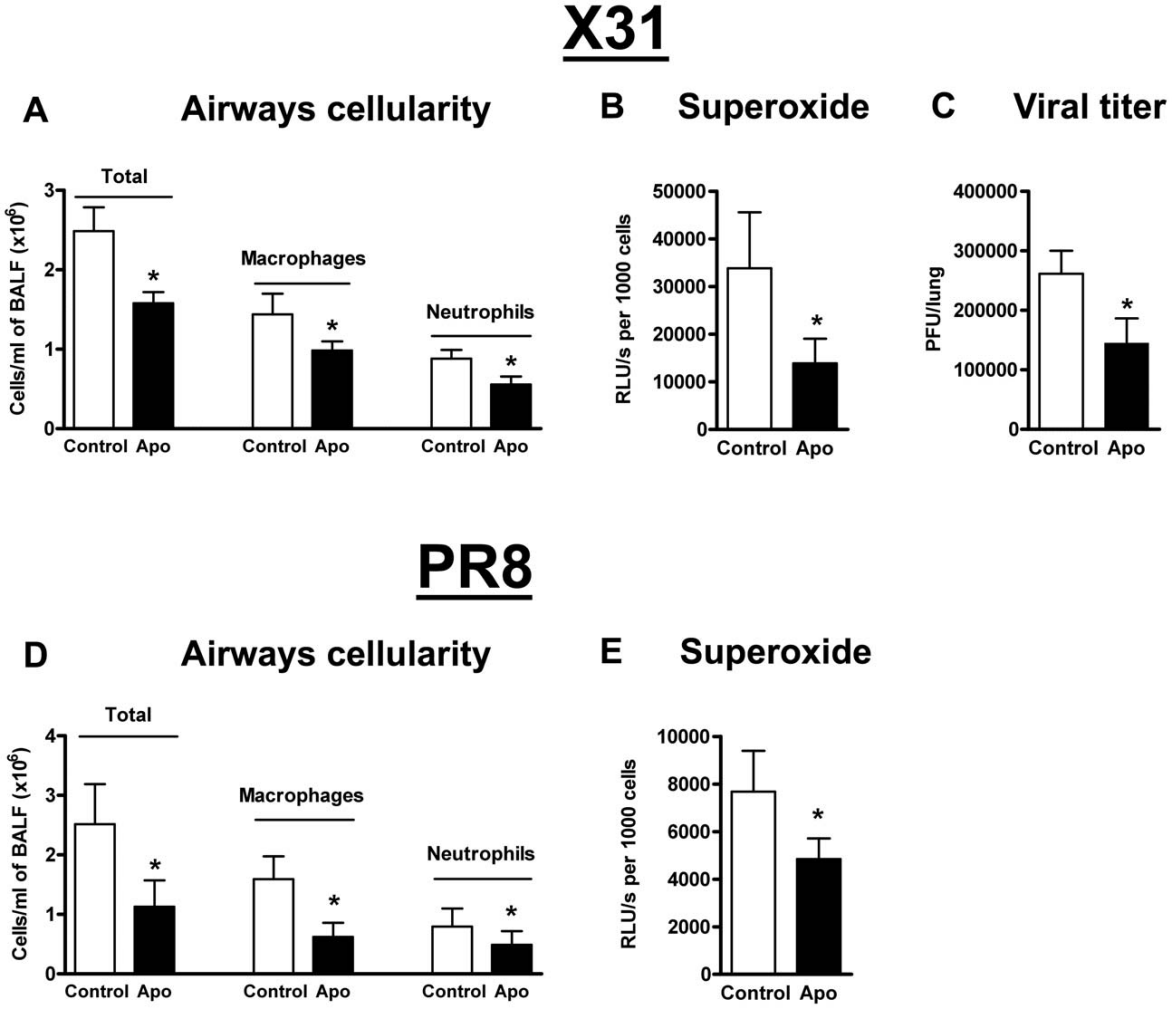
Pharmacological inhibition of Nox2 oxidase activity. Effect of apocynin (Apo, 2.5 mg/kg) treatment on (A) BALF cellularity, (B) BALF inflammatory cell superoxide production and (C) viral titer in H3N2 (X-31) influenza A virus-infected WT mice 3 days post infection. Also, effect of apocynin treatment on (D) BALF cellularity and (E) BALF inflammatory cell superoxide production in H1N1 (PR8) influenza A virus-infected WT mice 3 days post infection. Data are shown as mean ± SD for 6–8 mice per treatment group. *P<0.05 vs WT vehicle (Control)-treated mice (ANOVA followed by Dunnett's *post hoc* test for (A and D) but Students' unpaired *t* test used for B, C and E).

As genetic deletion of Nox2 suppresses lung injury associated with influenza A virus infection, we examined whether pharmacological inhibition of Nox2 *in vivo* with apocynin similarly protects against X-31 influenza A virus infection. Apocynin treatment of influenza A virus-infected WT mice resulted in significant reductions in BALF cellularity and numbers of macrophages and neutrophils (*P*<0.05) (Figure 9A). Basal and PDB-stimulated superoxide production were significantly lower ∼60% (*P*<0.05) in BALF cells isolated from apocynin-treated mice than in cells from untreated mice (Figure 9B). Strikingly, apocynin-treated mice displayed ∼50% reduction in viral titers when compared to untreated controls indicating that apocynin not only has anti-inflammatory properties but also facilitates virus clearance (Figure 9C).

We also examined the effect of apocynin treatment following PR8 influenza A virus infection in WT mice. As with X-31 infection, apocynin significantly suppressed BALF cellularity and superoxide production (Figure 9D–E).

Nitrite levels were similar in BALF taken from X-31 and PR8 virus-infected control (18.98±1.17 µM and 21.31±5.23 µM, respectively) and apocynin-treated mice (16.23±1.11 µM and 17.14±2.5 µM, respectively). (n = 4–7).

## Discussion

The recent outbreak of pandemic H1N1 influenza A virus has reinforced the urgent need for new pharmacological strategies that not only reduce virus replication but also the often-lethal, lung injury. Ideally, new therapies should show strong clinical efficacy against emerging and seasonal influenza A virus strains. The present study has shown that the absence of Nox2 (i.e. Nox2^−/y^ mice) or inhibition of Nox2 activity (apocynin-treated mice) substantially reduces inflammatory cell superoxide production, lung peroxynitrite levels, airways inflammation and apoptosis following infection with low and high pathogenicity influenza A virus strains. Strikingly, it also enhances clearance of the virus. Thus, modulation of Nox2 oxidase activity may represent a new therapeutic approach for controlling the lung inflammation caused by influenza A virus strains for which the population has no pre-existing immunity.

Snelgrove *et al.*, 2006 provided evidence for a significant improvement in lung function and for a reduced level of lung tissue damage in Nox2^−/−^ mice following low pathogenicity infection with X-31 influenza A virus [7]. In the present study we utilized a dose of X-31 that causes substantially greater BALF inflammation. We found that pulmonary challenge of wild type mice with X-31 resulted in a substantial degree of lung inflammation characterised by an accumulation of inflammatory cells around the peri-bronchial regions. By contrast the same viral infections caused markedly less peri-bronchial inflammation in Nox2^−/y^ mice. It is widely regarded that influenza A virus infection can cause extensive alveolar epithelial damage due to apoptosis resulting in alveolar leakage and consequently lung oedema and dysfunction [12]. In the present study, we show for the first time a substantial reduction in alveolar epithelial apoptosis in Nox2^−/y^ mice compared to WT controls. Previous studies examining the effects of infection with the highly lethal avian H5N1 virus in mice genetically deficient in the regulatory subunit of Nox2 activity, p47^phox^, demonstrated similar protective effects to those seen here in Nox2^−/y^ mice including significantly less pulmonary oedema [4]. Thus, Nox2-containing NADPH oxidase appears to be detrimental to the host following influenza A virus infections that cause mild, modest and even severe lung injury [4], [7].

The lethal lung pathology caused by pandemic strains of influenza A virus is believed to be due, at least in part, to an excessive host innate response characterized by a massive infiltration of macrophages and neutrophils [2], [13]. Activated macrophages and neutrophils produce large amounts of superoxide to limit the virus. However, in excess amounts, superoxide is toxic and capable of inducing significant injury to surrounding tissue. Indeed, this was exemplified by studies that showed that mice survived a potentially lethal influenza A virus infection when treated with pyran conjugated superoxide dismutase (SOD) to inactivate superoxide [3]. Although superoxide possesses considerable oxidising power to modify redox sensitive pathways and cause oxidative damage to cells, its biological toxicity is most likely due to its ability to rapidly give rise to a number of ROS and reactive nitrogen species with greater oxidising capacity such as OONO^−.^. These molecules have detrimental biological effects including nitration and oxidation of macromolecules, including proteins and DNA. Furthermore, it is well established that peroxynitrite is a powerful stimulant of apoptosis *via* the caspase 3 pathway (for review see [14]). Previously, it was concluded that one of the most important pathogenic factors in influenza A virus–induced pneumonia is peroxynitrite [15]. In the present study we show that the source of superoxide was solely Nox2 oxidase — as BALF cells isolated from Nox2^−/y^ mice produced almost no superoxide both basally and following stimulation with PDB. Moreover, we show *for the first time*, substantially less peroxynitrite in the lungs of Nox2^−/y^ mice compared to WT mice, indicating unequivocally that Nox2 is the major source of peroxynitrite induced by influenza A virus infection. Thus, it is highly likely that the protective effects of Nox2 inhibition are due to a reduction in superoxide and one of its major downstream derivatives, peroxynitrite.

Although from the present study, it appears that Nox2 is the predominant source of BALF inflammatory cell superoxide and the major source of lung tissue peroxynitrite production following influenza A virus infection, previous studies have implicated xanthine oxidase expressed in airways epithelium as a source of pathogenic superoxide [3]. Therefore, perhaps the most efficacious therapeutic approach against influenza A virus-induced lung oxidative stress would be to abolish the activities of *both* Nox2-containing NADPH oxidase and xanthine oxidase.

Pulmonary challenge with influenza A virus initiates a persistent infiltration of cells into the lungs, which marks the beginning of the innate immune response. Our study has shown that at 3 days post infection, the predominant cell types in the airway are the macrophages followed by neutrophils. By contrast, lungs of infected Nox2^−/y^ mice had generally less cells compared with wild type controls particularly at Day 7. Interestingly, this reduced airways cellularity in Nox2^−/y^ lungs appeared to be due to a specific reduction in macrophage numbers which were ∼40% lower than in wild type controls. The reason for a reduction in macrophage numbers in Nox2^−/y^ lungs is likely due to a reduction in their recruitment as levels of the chemotactic factor MCP-1 in these lungs were significantly lower. Indeed, studies performed with MCP-1 knockout mice showed a significant reduction in macrophage recruitment into the lungs during influenza infection [16]. MCP-1 production can occur in a number of cells including the airways epithelium. Although ROS are classically considered to cause lung damage when produced in excess because they are toxic to cells, they may also be involved in signal transduction pathways that regulate release of pathogenic cytokines such as MCP-1 from airways epithelium. Irrespective of the mechanism of ROS-dependent cytokine release, our study indicates that inhibition of Nox2-derived ROS has the potential to reduce the overall numbers of infiltrating inflammatory cells into the airways and hence the overall immunopathological clinical features induced by pandemic-inducing strains of influenza A viruses.

In this respect our study contradicts the Snelgrove et al. study in that macrophage numbers and overall inflammatory cell numbers were significantly lower in Nox2^−/y^ mice whereas Snelgrove et al., showed a heightened level of these cells. We hypothesize that differences may be attributed to the gender of the host i.e. male mice in our study as opposed to female mice by Snelgrove *et al.*, 2006 [7]. Although currently, we have no experimental evidence to suggest a potential gender difference in the airways inflammation caused by influenza virus infection, we have previously demonstrated a considerable gender difference in Nox2 activity under the settings of experimental stroke in mice [17]. Specifically, we showed that Nox2 oxidase activity in cerebral arteries is substantially higher in male mice compared to female mice [17]. A potential gender difference in the airways inflammation caused by influenza A virus infection certainly warrants further investigation.

Our study reveals a protective anti-inflammatory effect of Nox2 inhibition that could potentially modulate the fatal immune response triggered by pandemic influenza. That is, inhibition of Nox2 not only suppresses the production of toxic ROS by infiltrated macrophages and neutrophils but it also results in a reduction in the actual numbers of these cells in the airways. Although this would appear to be an attractive way of modulating an aggressive host immune response triggered by influenza A viruses, the dampening of this pro-inflammatory response might be predicted to compromise the ability of the host to effectively eliminate the virus. This has been well documented with the use of immunomodulators such as the COX2 inhibitors celecoxib and mesalazine, and for steroids. These compounds are effective at suppressing the early innate immune response but this comes with the cost of compromising viral clearance [18], [19]. In the present study, we showed that Nox2 deletion results in a reduction in lung viral titers. However, how inhibition of Nox2 derived ROS results in a ‘paradoxical’ reduction in virus titer in the lungs is unknown, for ROS are classically believed to be necessary for clearance of pathogens. One possibility is that the protective effect of superoxide inhibition is due to a resulting increase in bioavailability of anti-viral NO (for review see [20]). In the present study we show that NO levels in the BALF of Nox2^−/y^ mice are unlikely to be different to WT mice, at least after 3 days post infection. Whilst this appears surprising, it is consistent with previous reports that have shown unequivocally that NO levels *only* seem to increase in the lungs after 4 days post infection [15]. Thus, at least at Day 3 post infection where we observe a considerable improvement in outcome, NO is unlikely to be contributing to this protection. Snelgrove *et al* proposed the reduced virus titers were due to heightened macrophage numbers in the airways in the Nox2^−/−^ mice and presumably enhanced clearance of the virus by these cells [7]. As mentioned, in the present study we show a reduction in macrophages in the Nox2^−/y^ lungs, and thus, this is unlikely to explain the reduction in viral titers observed here. Although we currently have no experimental evidence to suggest how suppression of Nox2 results in a reduction in viral titers, one strong possibility is a reduction in virus replication within the airways epithelium. A number of studies have shown evidence that removal of ROS with antioxidants such as N-acetyl-cysteine suppresses replication of influenza A viruses including H5N1 in airway epithelial cells [21]. ROS are proliferative mediators and thus, may be crucial for viruses to replicate within cells. Overall, it appears that suppression of Nox2 and its production of ROS may have a dual beneficial effect, which includes suppression of lung apoptosis/inflammation and viral titers.

We also addressed whether inhibition of Nox2 influences important processes of the adaptive immune system. For example, does Nox2 oxidase play a role in antigen processing and presentation to CD8+ T lymphocytes? [22]. This process is crucial for effective activation of the adaptive immune response as it stimulates proliferation of CD8+ T lymphocytes that recognize viral antigens presented by infected cells. Snelgrove et al., 2006 showed a modest increase in CD8+ T cell numbers in the BALF of Nox2^−/−^ mice compared to WT mice and suggested this is likely to explain the enhanced viral clearance in this strain of mice. In the present study we quantified virus antigen specific CD8+ T lymphocytes in the spleen and BALF using tetrameric complexes targeting influenza-specific CD8+D^b^NP_366_+ and D^b^PA_224_+CD8+ T cells. Our findings indicate that in the absence of Nox2, antigen specific T cell-mediated immunity is preserved.

To demonstrate therapeutic relevance of our findings, we examined whether inhibition of Nox2 activity with apocynin similarly protects against influenza A virus infection. Over the past decade apocynin has gained recognition as the gold standard inhibitor of Nox2 activity by preventing the association of p47^phox^ with the Nox2 subunit (for review see [5]). There have been numerous reports describing a beneficial effect of apocynin in the settings of hypertension, cerebral ischemia-reperfusion injury, stroke outcome and myocardial infarction via its ability to inhibit Nox2 [5]. In the present study, mice were treated with apocynin at a dose (i.e. 2.5 mg/kg/day) shown previously to protect mice against ischemia damage caused by stroke [23]. Importantly, the effect of this dose of apocynin was similar to that seen in Nox2^−/y^ mice and apocynin did not provide further protection in Nox2^−/y^ mice, confirming a Nox2 specific action of apocynin [23]. Our study shows that administration of apocynin causes a powerful suppression of airways inflammation characterised by a decrease in the infiltration of macrophages, and neutrophils. This culminated in a substantial and visible reduction in oedematous bloody lung tissue following PR8 infection. In addition, apocynin significantly reduced virus titers. Thus, our study demonstrates an important and potentially clinically attractive effect of apocynin. That is, not only does apocynin possess potent anti-inflammatory effects but also it has the ability to promote viral clearance. Future studies will examine the use of apocynin, and other novel Nox2 inhibitors, as potential therapeutics.

To summarize, current strategies for the treatment of influenza A virus-induced lung disease are focussed primarily at halting mechanisms of viral infection and replication [24]. Far less attention has been directed to investigating mechanisms that modulate host responses, which lead to lung inflammation and pathology. In the present study we evaluated the role of Nox2 oxidase, the primary source of ROS in immune cells in the lung immunopathology caused by influenza A virus of varying virulence. The absence of Nox2 reduced airways inflammation, lung oxidative stress and apoptosis. Moreover, Nox2 may impede with virus clearance and/or stimulate virus replication. We envisage that use of Nox2 inhibitors, in combination with antiviral strategies, may be effective tools against morbidity and mortality induced by seasonal and pandemic stains of influenza A virus.

## Materials and Methods

### Animal breeding

Specific pathogen-free male C57BL/6 (wild type) and Nox2 deficient (Nox2^−/y^) mice (C57BL/6 background) aged between 7 and 10 weeks and weighing ∼25 g were bred at the Department of Pharmacology at Monash University. The animals were housed at 20°C on a 12 h day/night cycle in sterile micro-isolators and fed a standard sterile diet of Purina mouse chow with water allowed *ad libitum*.

### Animal ethics statement

The experiments described in this manuscript were approved by the Animal Experimentation Ethics Committee of The University of Melbourne and conducted in compliance with the guidelines of the National Health and Medical Research Council (NHMRC) of Australia on animal experimentation.

### Infection of WT and Nox2^−/y^ mice

Male naïve WT control and Nox2^−/y^ mice were anaesthetized by penthrane inhalation and infected intranasally (*i.n.*) with 1×10^4^ plaque forming units (PFU) X-31 (H3N2) or 50 PFU PR8 (H1N1) in a 35 µl volume, diluted in PBS. All mice were killed at day 3 (peak of viral titres) or day 7 (resolution of infection) following influenza infection.

### In vivo pharmacological inhibition of Nox2

The Nox2 selective inhibitor, apocynin (2.5 mg/kg) was administered to WT mice via intraperitoneal (*i.p.*) injection daily for 3 days prior to either X-31 or PR8 influenza A virus infection, and for up to 3 days following infection. We have previously shown that 2.5 mg/kg apocynin can selectively inhibit Nox2 oxidase activity [23].

### Airways inflammation and differential cell counting

Mice were killed by an i.p. injection of sodium pentobarbitone (360 mg/kg) and BAL performed as we have previously shown [25], [26]. Briefly, lungs from each mouse were lavaged *in situ* with a 400 µl aliquot, followed by three 300 µl of PBS. The total number of viable cells in the BALF was determined by using ethidium bromide and acridine orange (Molecular Probes, San Diego, USA) on a standard Neubauer hemocytometer using a Zeiss Axioscope Fluorescence microscope. Cytospins were prepared using 20–50 µl BALF at 350 rpm for 10 min on a Cytospin 3 (Shandon, UK). Cytospin preparations were stained with DiffQuik (Dade Baxter, Australia) and 500 cells per slide were differentiated into macrophages, neutrophils, eosinophils, and lymphocytes by standard morphological criteria. The remaining BALF was spun at 3000 rpm for 5 min to pellet cells for flow cytometry and superoxide detection. In addition, the spleens of virus-infected mice were collected for flow cytometry.

### Histology

Histology was performed as previously described [26]. Briefly, mice were killed by i.p. anaesthesia (360 mg/kg sodium pentobarbitone) overdose and then perfusion fixed via a tracheal cannula with 10% neutral buffered formalin at exactly 200mm H_2_O pressure. After 10 min, the trachea was ligated, the lungs were removed from the thorax and immersed in 10% neutral buffered formalin for 24 h. After fixation of the lung tissue and processing in paraffin wax, sections (3–4 µm thick) were cut longitudinally through the left and right lung so as to include all lobes. Sections were stained with hematoxylin and eosin (H&E) for assessment of general histopathology.

### Superoxide detection with L-O12 enhanced chemiluminescence

BALF inflammatory cells were exposed to the chemiluminescent probe, L-O12 (100 µM; Wako laboratories, Japan) in the absence or presence of the PKC and NADPH oxidase activator, phorbol dibutyrate (1 µM; Sigma) and dispensed into 96 well white opti-plates for luminescence reading with the TopCount (Packard). Photon emission was recorded from each well every 2 min and averaged over 45min. Individual data points for each group were derived from the average of 3 replicates. In some cases, cells were incubated with superoxide dismutase (SOD; 600U/ml) to inactivate superoxide and to verify that the L-O12 chemiluminescence signal was due to superoxide.

### Immunohistochemistry

Lung sections from WT and Nox2^−/y^ mice infected with X-31 were deparaffinised and fixed in acetone for 15 min. Tissues were then washed in 0.01 M phosphate buffered saline (PBS, pH 7.4; 3×10 min) before incubation in a Mouse on Mouse Ig blocking reagent (Vector Laboratories) for 1 h to reduce non-specific binding. Tissue mounted sections were incubated in either mouse monoclonal anti-3-nitrotyrosine (1∶50, AbCAM) or rabbit polyclonal anti-cleaved caspase 3 antibody (1∶250, AbCAM) overnight in a humid box. The following day, tissues were washed in 0.01 M PBS (3×10 min) to remove any excess antibody, and incubated in a biotinylated anti-mouse IgG reagent for 10 min for 3-nitrotyrosine studies or a goat anti-rabbit Alexa fluor 488 (Invitrogen; 1∶500) secondary antibody for caspase 3. Lung sections were then washed in 0.01 M PBS (3×10 min) and Fluorescein Avidin DCS (Vector Laboratories) was applied for 5 min. Sections were washed in 0.01 M PBS (3×10 min) and cover slipped. Slides were viewed and photographed on an Olympus fluorescence microscope. Researchers were blinded throughout the experiment and all the appropriate primary and secondary controls were performed.

### Viral titres

Lungs from influenza virus-infected mice were removed, weighed and homogenised in 2 ml of RPMI medium 1640 containing 24 µg/ml gentamycin and 100 units/ml penicillin/streptomycin. Viral titres (PFU/ml) were determined by plaque assay on Madin-Darby Canine Kidney (MDCK) cell monolayers as previously published [27].

### Griess reaction for nitrite determination

NO levels in BALF were determined by quantifying its product, nitrite, using the Griess reaction kit according to the manufacturer's instructions (Molecular Probes). Nitrite levels were determined from 150 µl of BALF, which was obtained from WT and from either Nox2^−/y^ or apocynin (2.5 mg/kg/day) treated mice killed Day 3 post X-31 or PR8 infection.

### Quantitative real-time PCR

Whole lungs were perfused free of blood via right ventricular perfusion with 10 ml of pre-warmed saline, rapidly excised en bloc, blotted and snap frozen in liquid nitrogen. Total RNA was extracted from 15 mg of whole lung tissue pooled from 5 mice per treatment group using RNeasy kits (Qiagen), reverse transcription with SuperScript III (Invitrogen) and triplicate real time PCR reactions with Applied Biosystems pre-developed assay reagents and 18S rRNA internal control were done as previously described [25], [26].

### Tetramer and intracellular cytokine staining

The numbers of BALF and spleen influenza-specific CD8+D^b^NP_366_+ and D^b^PA_224_+ T cells was assessed using tetramer and intracellular cytokine staining as we have previously described. Splenocyte suspensions were initially depleted of B cells by panning on 150-mm petri dishes coated with goat anti-mouse IgG/IgM (Jackson ImmunoResearch, CA, USA) for 1 h at 37°C. BALF was placed on plastic for 1 h at 37°C to remove macrophage populations. Enriched CD8+ lymphocyte populations from the spleen and BALF were then stained with fluorescently labelled tetrameric complexes directed against two immunodominant influenza-specific CD8+ T cells epitopes (D^b^NP_366–372_ or D^b^PA_224–232_) to determine the numbers of the responding population. In addition, enriched CD8+ T cells were incubated for 5 h in 96-well round bottom plates with 1 mM influenza NP_366–372_ or PA_224–232_ peptide in the presence of Brefeldin and IL-2 and then fixed and stained for the presence of CD8α, IFN-γ, TNF-α and IL-2 as previously described [28]. Data was acquired on a BD FACS Calibur (Becton Dickinson, USA) and analysed using Cell Quest Pro software.

### Statistical analyses

As data were normally distributed, they are presented as grouped data expressed as mean±standard deviation of the mean (SD); *n* represents the number of mice. Differences in BALF cell types and whole lung mRNA expression were determined by analysis of variance (ANOVA) followed by Dunnett *post hoc* test for multiple comparisons, where appropriate. In some cases, Student's unpaired *t*-test was used to determine if there were significant differences between means of pairs. All statistical analyses were performed using GraphPad Prism for Windows (Version 5.0). In all cases, probability levels less than 0.05 (*P<0.05) were taken to indicate statistical significance.
